# Supplementary figures and images for: Giardia lamblia risk factors and burden in children with acute gastroenteritis in a Nicaraguan birth cohort
Source: PLoS Negl Trop Dis. 2024 Nov 11;18(11):e0012230. doi: 10.1371/journal.pntd.0012230 (PMC11581391; doi:10.1371/journal.pntd.0012230)

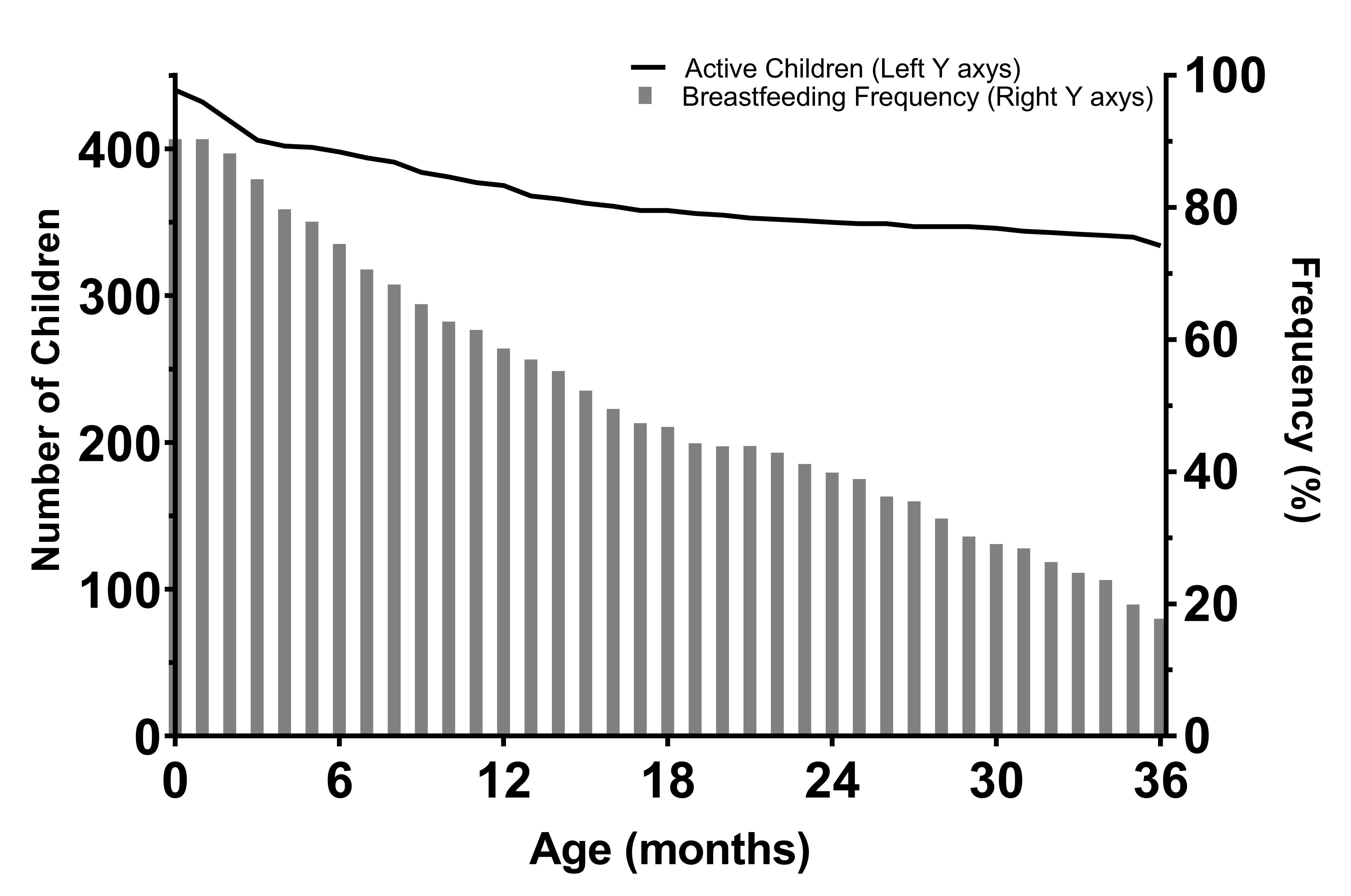

Supplement: S1 Fig — (TIF) [file pntd.0012230.s002.tif]

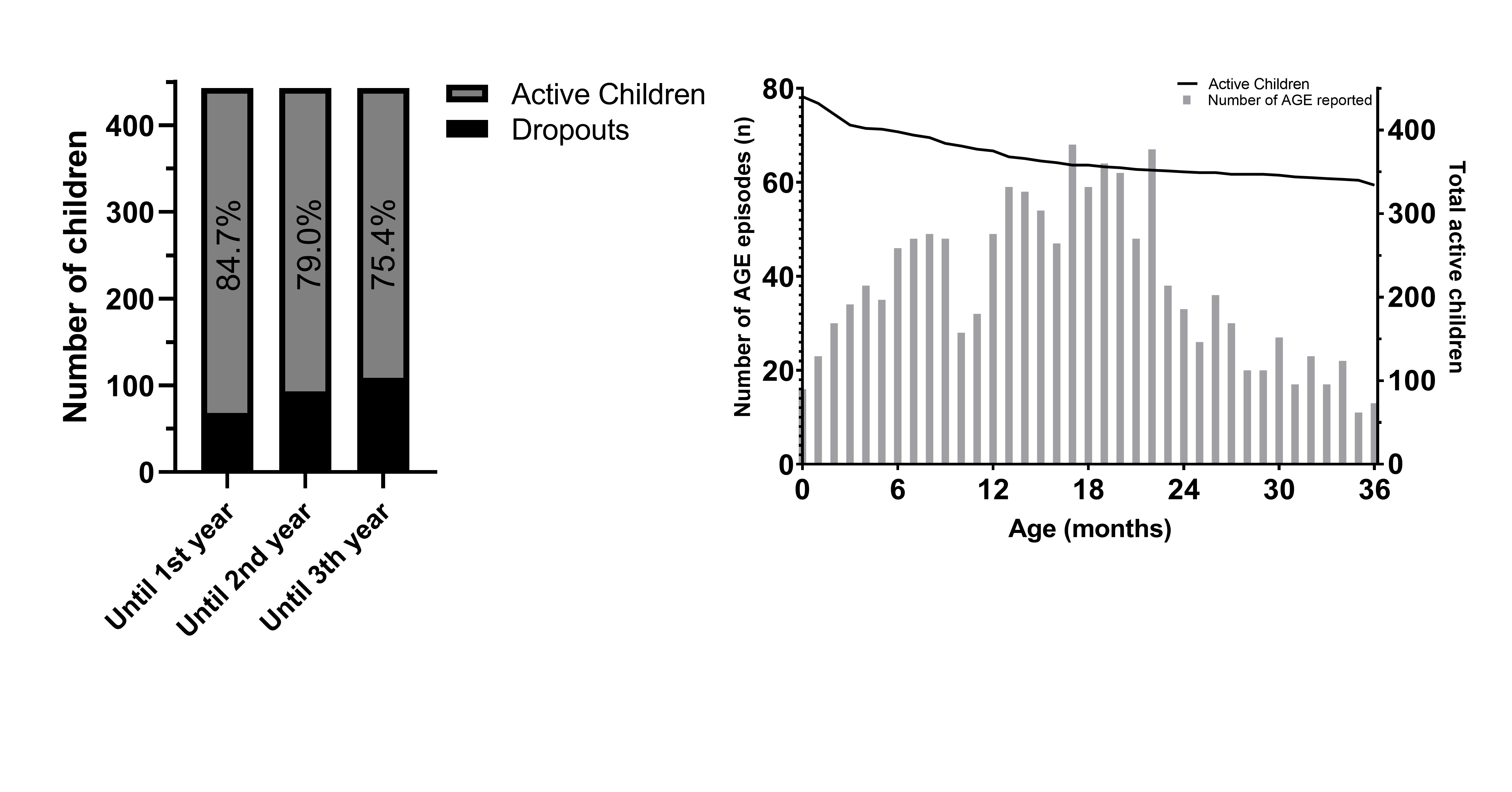

Supplement: S2 Fig — A) Frequency and retention rate over the cohort by year. B) Number of monthly acute gastroenteritis episodes (AGE) reported (gray bar, left Y axis) and total number of active children by age in months (black line, right Y axis). (TIF) [file pntd.0012230.s003.tif]
