# Supplementary material for: Giardia lamblia risk factors and burden in children with acute gastroenteritis in a Nicaraguan birth cohort
Source: PLoS Negl Trop Dis. 2024 Nov 11;18(11):e0012230. doi: 10.1371/journal.pntd.0012230 (PMC11581391; doi:10.1371/journal.pntd.0012230)
Supplement: Attachment — Submitted filename: Reviewer_Response_080924.docx [file pntd.0012230.s005.docx]

**PLOS Neglected Tropical Diseases submission (PNTD-D-24-00714) –**

**[EMID:9588b83a6dc9e1df]**

**Title of Article:** *Giardia lamblia* risk factors and burden in children with acute gastroenteritis in a Nicaraguan birth cohort.

**Name of the Corresponding Author:** Nadja A. Vielot

**Email:** nadjavielot@unc.edu

**Dear Paul Brindley**

**Editor-in-Chief**

**PLOS Neglected Tropical Diseases**

We appreciate and generally agree with the reviewer's comments summarizing some challenges.

We re-wrote the title to focus on risk factors for GAGE as the main aim of this study. We consider the importance of studying GAGE to first step to guide efforts to reduce the burden of disease in this region of Central America, where *Giardia* studies have been neglected for more than a decade. We also recognize the importance of studying asymptomatic infections, which we believe should be incorporated into future research efforts to better understand the full spectrum of the infections.

The grammar text has been extensively reviewed and English grammar and composition were adjusted accordingly. Also, we applied some changes according to the reviewer's comments detailed below.

--------------------

**Reviewer Responses to Questions**

**Reviewer #1**

- Missing asymptomatic giardiasis is a huge limitation to the current works making interpretation very difficult.

R: We agree that missing asymptomatic *Giardia* infections is one limitation for this study declared in the discussion section. Despite this limitation, we consider the importance to study the risk factors associated with *Giardia* during acute gastroenteritis in order to guide efforts to reduce the burden of disease. León, Nicaragua is an endemic area where the most recent study that includes *Giardia* dates back more than a decade, prior to widespread use of molecular methods for *Giardia* detection.

- Descriptive statistics of the missing 113 diarrheal episodes (or the reason in which weekly sampling couldn’t collect a sample within 10 days) would be helpful in determining if there is bias here.

R: The main reason stools were missing was logistical challenges in collecting the stool on time (the mom would throw it away, was uncomfortable keeping it in the fridge, unsafe to be in the field during the political unrest and COVID pandemic) and stools are likely missing at random. We have used imputation to correct for missing stools in a previous study and did not find substantial changes in sapovirus incidence, thus we think the missing samples do not cause substantial bias in our estimates: <https://www.ncbi.nlm.nih.gov/pmc/articles/PMC7878336/>

- A CT <37 seems high.  Can authors give an explanation of how this cutoff for positivity was chosen?
R: Thank you for this observation, it was a typing error. The correct cutoff used was fixed ≤35. This Ct value was chosen according to different published studies:

<https://www.nature.com/articles/s41467-023-38363-2>

<https://journals.plos.org/plosone/article?id=10.1371/journal.pone.0158199>

<https://www.sciencedirect.com/science/article/abs/pii/S1473309914708084>

- How many children were excluded for having <6months of follow up?
R: 45 children, now is added in line 185

- It is unclear which variables were included in the multivariate models?  All variables listed in table 3 or just those significant in univariate analysis?
R: Added in line 175-177: “A multivariable analysis was performed using significant variables in crude analysis and was adjusted including potential confounders for each variable”

- Methods detailing the collection of all of the data that makes up table 3 needs to be included in the methods.  For instance, how was presence of mice in the home determined?
R: Added in line 106-113: “Furthermore, epidemiological characteristics were collected at baseline and updated at each study visit, mothers were asked whether they had breastfed their child the previous day and whether the child had consumed anything besides breast milk in the past week. Additionally, each month, mothers were asked to provide extensive risk factor data regarding household conditions and interpersonal contact, including factors that were unlikely to change on a weekly basis (e.g., water treatment measures, water storage, and the presence of animals in the household), Those variables collecting are self-report and they could be subject to reporting bias”

- Data on the quantity or frequency of breastfeeding would be helpful as so few mothers were exclusively breastfeeding and breast milk seems protective.
R: Agreed. We reported the median (IQR) for exclusive and non-exclusive breastfeeding in Table 1, but to support that information, we have now also added a supplementary figure S1 showing the monthly breastfeeding frequency.

**Reviewer #2**

- Study design is appropriate but, exclusion of other major intestinal infections has not been done. Sample size is sufficient and correct analysis has been done.

R: We agree that missing other major intestinal infections (including other *Giardia* outcomes like asymptomatic infections) is one limitation for this study. Despite this limitation, we consider the importance to study the risk factors associated with *Giardia* during acute gastroenteritis in order to guide efforts to reduce the burden of disease.

**Reviewer #3**

-Are the objectives of the study clearly articulated with a clear testable hypothesis stated?
Yes, it is good to make a study of this type in order to see what the effetcs are of Giardia infections in children in this region of the world.

-Is the study design appropriate to address the stated objectives?
Yes, it is. As the authors state it would be good to look at asymptomatically Giardia infected children as well and do genotyping.

R: We agree that missing asymptomatic *Giardia* infections and the lack of genotyping are limitations of this study. However, we still believe it is important to investigate the risk factors associated with GAGE. This research can help inform efforts to reduce the disease burden in this endemic area.

-Is the population clearly described and appropriate for the hypothesis being tested?
Yes
-Is the sample size sufficient to ensure adequate power to address the hypothesis being tested?
Yes
-Were correct statistical analysis used to support conclusions?
Yes
-Are there concerns about ethical or regulatory requirements being met?
No

--------------------

**Results**

**Reviewer #1**

-3 weeks of exclusive breast feeding is extremely short in a LMIC setting.  It would be helpful for the reader to understand if this was being supplemented with formula and, if so, the water source of households becomes very important to this study.
R: Unfortunately, we do not have this specific information. We have data on water sources, and we have data on supplemental feeding, but we did not ask about the content of supplemental foods, i.e. we don’t know if they were using formula.

The way we asked the question was: During the past week, has your child received any food, formula, or liquid, including water or tea or pacifier, other than breast milk? Yes / No / Refused to answer

-In figure 1 it would be helpful to have the sample size by month on the figure or some discussion of attrition so that the reader understands the decrease in GAGE during the 3rd year of life is not due to less children enrolled.
R: Agreed, we consider this comment important. We have added information about the retention time of the cohort by year, as well as a supplementary figure S2 on retention rates. Please see lines 195-197:

“By the end of the cohort, 109 children had dropped out (retention rate of 75.4%), with the highest dropout rate occurring during the first year (68/443 children) (Figure S2).”

-Discussion of testing for other pathogens should be moved to the methods section.  Why were only 76/104 GAGE episodes tested?  Which other pathogens were assayed for and the methods need to be detailed.
R: The methodology used for the co-pathogens molecular detection was described in methods section. Please see lines 153-159:

“Seventy-six GAGE episodes were tested by qPCR for 13 other common enteric pathogens using oligonucleotide primers described by Liu J *et al*[25] in the multiplex qPCR platform. These included rotavirus, adenovirus, astrovirus, norovirus, sapovirus, Enterotoxigenic *Escherichia coli* (ETEC), Enteropathogenic *E. coli* (EPEC), Enteroinvasive *E. coli*/Shiguella (EIEC/Shiguella), Enteroaggregative *E. coli* (EAEC), Shiga Toxin-producing *E. coli* (STEC), *Campylobacter* spp, *Entamoeba histolytica*, and *Cryptosporidium* spp.”

Additionally, we removed the description for 13 other pathogens tested by qPCR in 76 GAGE episodes as was suggested (line 212) and moving to methodology as describe before.

Out of the 104 GAGE episodes, only 76 were tested due to sample availability, as many of the samples were shared with other studies. However, we believe that the data obtained from the 76 processed samples provide valuable insights into the presence of other enteric pathogens during the GAGE episodes.

-Lines 202-205:  This is not clear?  Was duration longer without co-infection?  It a poorly written sentence grammatically which obscures meaning.

R: It was re-written in lines 226-229: “Similarly, no differences in clinical characteristics were found when comparing GAGE co-infected with another pathogen versus GAGE without co-infection. However, the duration of diarrhea in GAGE tended to be longer in the absence of other etiologies (median of days of diarrhea [IQR]: 7 [3-11] vs 4 [3-7]) (S1 Table).”

Additionally, it was included as part of the discussion section (added in lines 289-299): “Although we did not find differences in clinical characteristics between GAGE with and without co-infections, the duration of diarrhea tended to be longer in the absence of other etiologies. *G. lamblia* is more associated with prolonged/persistent diarrhea[3] and the impact of GAGE, in the presence of other etiologies, on the clinical characteristics and persistence of diarrhea is a critical area of research, especially in populations with high exposure to multiple enteric pathogens. Co-infections with *Giardia* and other pathogens do not generally increase the severity of diarrhea across all populations. The interaction *G. lamblia* with other pathogens can influence clinical outcomes; for example, co-infection with rotavirus and *G. lamblia* was found to reduce the severity of rotavirus-induced diarrhea[33]	(https://link.springer.com/article/10.1023/B:EJEP.0000040533.75646.9c). Moreover, *G. lamblia* can modulate host immune responses, potentially affecting the clinical outcomes of co-infections, which may influence the severity of AGE[34] (<https://www.mdpi.com/2076-0817/4/4/764>). Further research is needed to fully understand these complex interactions and their implications for clinical outcomes during GAGE.”

-Please list p values in table 3
R: We followed the authorship guidelines from PLOS NTDs:

*“Describe statistical methods with enough detail to enable a knowledgeable reader with access to the original data to judge its appropriateness for the study and to verify the reported results. When possible, quantify findings and present them with appropriate indicators of measurement error or uncertainty (such as confidence intervals). Avoid relying solely on statistical hypothesis testing, such as P values, which fail to convey important information about effect size and precision of estimates.”* <https://journals.plos.org/plosntds/s/submission-guidelines>

-Lines 233 – 239 need to moved to the methods section.  They also need to be revised for clarity.  The model that was created (and why the covariates were chosen as they were) should be clarified.  It seems several models were created?
R: Lines 233-239 were moved to the methods section as suggested and were also revised to better clarity and understanding.

Models and covariables chosen are described in lines 177-181: “to determine whether breastfeeding duration has a protective impact on reducing GAGE in children living in a high-risk population characterized by household conditions variables that were statistically significant in the crude analysis (no indoor toilet, earthen floor, and presence of mice), we use breastfeeding duration according to the number of weeks the mother reported any breastfeeding, exclusive or not, in the first 6 months.”

-Results of the breast feeding analysis are confusing.  Presence of an indoor toilet is used in two of the models (one alone and one in the subset that also had an earthen floor)?  Why? How do you interpret this when earthen floor is not significant?
R: Models were created using household conditions variables that were statistically significant in the crude analysis and have also been commonly reported as risk factors in the literature and associated with poverty (no indoor toilet, earthen floor). We also included, as an exploratory analysis, the presence of mice (highest HR in our crude analysis) and the joint interaction of both no indoor toilet and having earthen floor with breastfeeding duration, according to the number of weeks the mother reported any breastfeeding, exclusive or not, in the first 6 months, to reduce the incidence of GAGE. Although the earthen floor alone was not significant in the model, the joint interaction of earthen floor and no indoor toilet had the highest HR in those models, even higher than the effect of no indoor toilet alone. This suggests that the joint presence of both variables is greater than their individual effects.

**Reviewer #2**

1.Abdominal pain and cramp is an important clinical characteristic features of AGE and G. Lamblia. According to one study , G. lamblia significantly contributes to recurrent abdominal pain in children (Buch N.A., Ahmad S.M., Ahmed S.Z., Ali S.W., Charoo B.A., Hassan M.U. Recurrent abdominal pain in children. Indian Pediatr. 2002; 39:830–834.). This was not mention in the  because their main aim is to describe the clinical characteristics.

R: Our first aim was to describe the risk factors for GAGE in order to guide efforts to reduce the burden of disease in this population. A secondary aim was to determine the epidemiological and clinical characteristics of GAGE updates. We use samples and data from the Sapovirus-Associated Gastro-Enteritis (SAGE) birth cohort study from Nicaragua which unfortunately abdominal pain and cramps were not included in clinical characteristics, as we were concerned with symptoms that could occur in a variety of pathogen-specific AGE episodes. In addition, symptoms were self-reported by the child’s mother or caregiver, and thus we did not collect subjective child-reported symptoms like cramps or pain.

2.How long do the symptoms persist in case of AGE with positive with G. lamblia? As the symptoms are mild and moderate, do not require medication mostly and which could increase the parasitic burden lead to chronic carriage.

R: Unfortunately, we do not have days of duration of symptoms. Only days of diarrhea were described in this study in Table 1 and Table S1.

3.The study did not compare the differences between malnourished vs normal children who are suffering from AGE associated with G. Lamblia? A study conducted in Pakistan indicate that between 9% and 10% of population has G. lamblia, with malnourished children under 10 years of age particularly affected. (Al-Mekhlafi M.H., Azlin M., Nor Aini U., Shaik A., Sa’iah A., Fatmah M.S., Ismail M.G., Ahmad Firdaus M.S., Aisah M.Y., Rozlida A.R., et al. Giardiasis as a predictor of childhood malnutrition in Orang Asli children in Malaysia. Trans. R. Soc. Trop. Med. Hyg. 2005; 99:686–691. doi: 10.1016/j.trstmh.2005.02.006)
R: A previous study from this cohort has evaluated malnourished kids, and prior analyses show that the kids are all very “well fed” (Table 2:

<https://www.ncbi.nlm.nih.gov/pmc/articles/PMC7878336/>). This was an exclusion criterion (healthy birth weight, no chronic diseases) from the birth cohort, and malnutrition as a risk factor for GAGE was not evaluated. Similarly, giardiasis as a predictor of childhood malnutrition was not included due to our limitation of reporting only GAGE and not asymptomatic infections. Recent data in children suggest that just the presence of *G. lamblia* (independently of symptoms) is associated with decreased growth velocities (<https://pubmed.ncbi.nlm.nih.gov/28204556/>). For that reason, we focus only on risk factors to reduce the burden disease.

4.Study did not show any relationship poverty, family size and income? But these are also a risk factor for G. lamblia.

R: Thank you for your observation. While the study did not include a direct poverty index as a risk factor, we did incorporate variables commonly associated with poverty, which are also used to calculate poverty indices according to references by Peña (<https://ajph.aphapublications.org/doi/10.2105/AJPH.90.1.64>) and Economic Commission for Latin America and the Caribbean (ECLAC/CELAC) (<https://www.mef.gob.pe/contenidos/pol_econ/documentos/Metodo_de_NBIs.pdf>) in this region. Specifically, the type of flooring (earthen floors) and lack of an indoor toilet are two of the five criteria used to define poverty in Nicaragua (the others being overcrowding, income, and education level). Additionally, in this study, the presence of mice in the household was used as an indicator of poor sanitary conditions and poverty. Therefore, Figure 3 of the article is related to variables typically associated with poverty. Although family size and income were not directly analyzed as risk factors, the variables included in the study do reflect important aspects of socioeconomic conditions.

**Reviewer #3**

-Does the analysis presented match the analysis plan?
Yes.
-Are the results clearly and completely presented?
Yes, well-presented.
-Are the figures (Tables, Images) of sufficient quality for clarity?
Yes

--------------------

**Conclusions**

**Reviewer #1**

-Again, is there evidence for the claim that not seeking care leads to prolonged shedding and sequelae?
R: It was rewritten in lines 281-287 as a possibility:

“We consider that the lack of care seeking for GAGE episodes could be important in the epidemiology of GAGE. If children do not seek medical attention, they may not receive a diagnosis of GAGE, potentially resulting in a lack of treatment. This absence of intervention could allow the parasite to persist, which might lead to chronic episodes. The persistence of *G. lamblia* is commonly associated with long-term sequelae[13,31,32]. Additionally, we found that the lack of access to indoor toilets and breastfeeding for less than 6 months are risk factors for GAGE.”

-Line 266:  Earthen floor was not associated with GAGE as reported above.  Please clarify.
R: It was a typing mistake and we removed it. Earthen floor was not associated with GAGE by itself.

-Discussion of why breastfeeding in the 2nd year of life is not protective when almost 50% of women continued to do it would be helpful.
R: Right! 55% of children continued to breastfeed for more than 12 months, but only 38% of children in the cohort reached the full two years of breastfeeding (this information is now available in supplementary figure S1). Additionally, the reduced protection from breastfeeding after the first year could be due to children not receiving enough breastmilk to be protective, or because they are sufficiently exposed to unsafe foods or environmental conditions, which may override the benefit of breastmilk. Moreover, a Bangladesh cohort study did not find an association between levels of anti-*Giardia* breastmilk and diarrhea from *Giardia* or the occurrence of repeat infections (<https://academic.oup.com/cid/article/56/7/988/359369>). We prefer to focus on the protective effect of early breastfeeding and encourage future studies on the role of breastfeeding throughout the first years of life (Line 336-339):

“…data supports the need for future qualitative and biological research to understand if breastmilk components such as IgA antibodies are important or whether the impact of breastmilk on microbiome composition may play a protective role in G. lamblia pathogenesis throughout the first years of life.”

-I don’t think the authors can state that any queried variable is “not associated with Giardia infection” as they only tested symptomatic infections and the majority of cases are asymptomatic.  Lines 299 – 303 are an example.
R: We re-wrote in this section lines 343-349:

“…suggesting that the presence of those antigens, in the intestinal epithelia or secretions might not be associated with GAGE, as observed for some viral and bacteria gastrointestinal infections. Although genetic susceptibility was not associated with GAGE in this study, more studies are needed to understand if those antigens play a role during *G. lamblia* infections, not only in the development of GAGE but also in establishing colonization in asymptomatic events. The genetic susceptibility at individual/host levels is a topic of ongoing research.

**Reviewer #2**

-Are the conclusions supported by the data presented?

Yes, conclusion  is supported   by the  data  presented.  It would  have been  nice if other  socioeconomic  conditions  have  been taken into account for analysis.

R: Agree! While the study did not include a direct poverty index as a risk factor, we did incorporate variables commonly associated with poverty, which are also used to calculate poverty indices according to references by Peña (<https://ajph.aphapublications.org/doi/10.2105/AJPH.90.1.64>) and Economic Commission for Latin America and the Caribbean (ECLAC/CELAC) (<https://www.mef.gob.pe/contenidos/pol_econ/documentos/Metodo_de_NBIs.pdf>) in this region, like lack of indoor toilet and earthen floors.

**Reviewer #3**

-Are the conclusions supported by the data presented?
Yes
-Are the limitations of analysis clearly described?
Yes, also discusssed in the Discussion part.
-Do the authors discuss how these data can be helpful to advance our understanding of the topic under study?
Yes.
-Is public health relevance addressed?
Yes.

--------------------

**Editorial and Data Presentation Modifications**

**Reviewer #1**

The grammar and sentence structure (mostly run-on sentances) is poor in places which obscures meaning.
R: The text has been now extensively reviewed and English grammar and composition adjusted accordingly

**Reviewer #2**

**(No response)**

**Reviewer #3**

Line 28. “200 million symptomatic  infections”

R: Done

Line 32, Explain AGE

R: Done

Line 72. “G. lamblia detection does not appear to be associated with moderate-to-severe diarrhea”, this is true in low income countries but in high income countries it is associated with diarrhea.

R: Added in line 75-77

“Instead, in developed countries, this parasite has been reported in clustered daycare outbreaks[6] and is characterized by seasonal and recreational waterborne transmission[7,8]. Additionally, it is one of the most common causes of diarrhea in travelers[9].”

Line 167. 45.4% born by cesarean section. High or normal int his environment?

R: It is normal. Scheduled cesarean section is common in LMIC with rates increasing over time. The latest available data (2010-2018) from 154 countries covering 94.5% of the world's live births shows that 21.1% of women gave birth by cesarean worldwide, averages ranging from 5% in sub-Saharan Africa to 42.8% in Latin America and the Caribbean. <https://www.ncbi.nlm.nih.gov/pmc/articles/PMC8208001/>

--------------------

**Summary and General Comments**

**Reviewer #1**

Authors have performed a secondary analysis to determine risk factors for gastroenteritis with Giardia detected and to determine the effects of breastfeeding on such episodes.  The cohort was designed to study Sapovirus and thus did not include asymptomatic stool collection as the majority of Sapovirus infections are symptomatic.  This is a major limitation of the current work as the majority of Giardia infections are asymptomatic.   This makes interpretation and extrapolation of the presented findings extremely difficult.  Identified “cases” are likely a vast under-representation of the burden of Giardia infection.  That said, the findings are relatively consistent with the literature on risk factors for Giardia and the protective effect of breast feeding.  This study does not add greatly to the literature as similar findings are thoroughly reported in the literature.

R: We agree that missing other major intestinal infections (including other *Giardia* outcomes like asymptomatic infections) is one limitation for this study. Despite this limitation, we consider the importance to study the risk factors associated with *Giardia* during acute gastroenteritis in order to guide efforts to reduce the burden of the disease, especially considering that the last study in León, Nicaragua, found *Giardia* to be associated with diarrhea (Table 2:	<https://pubmed.ncbi.nlm.nih.gov/24879131/>). Although our findings are consistent with the existing literature, this is a new geographical area. Epidemiology and outcome variations have even been observed in multi-site studies following the same enrollment criteria, protocol, measurements, and analyses, suggesting that study design is not the primary driver of the observed outcome variability (MAL-ED cohort study). Although breastfeeding has been previously reported as protective, our work quantifies this effect and its temporality in a specific population with risk factors associated with GAGE, which could inform the design of future interventional studies. We recognize the importance of studying asymptomatic infections, which we believe should be incorporated into future research efforts to better understand the full spectrum of the infections.

**Reviewer #2**

**As above**

**Reviewer #3**

This study essentially verifies what has already been shown in earlier studies of Giardia infections in children. However, this is a new region and setting and the conclusions are clear.
Genotyping of the GAGE samples would have been good to resolve issues of animal/assemblage infections and it could have been correlated to other data.

R: We agree that the genotyping of GAGE samples could help resolve issues of animal/assemblage. Unfortunately, it was not performed, and we consider the risk of the presence of mice as an indicator of poor sanitary conditions. See lines 310-317:

“Interestingly, in our cohort, GAGE was more common in households with presence of mice. While some *G. lamblia* strains can persist in experimental murine models [43], there is not evidence that mice are a reservoir for humans. Mice naturally carry *G. muris* and/or different *G. lamblia* genotypes (assemblage G)[40]; unfortunately, the genotyping of GAGE samples was not performed in this study. While observational studies are needed to investigate if mice can be infected with *G. lamblia* assemblages A and B to contribute to local endemicity, here we consider that the presence of mice at home is most likely be an indicator of poor sanitary conditions in the household.”

--------------------

Other changes:

- The affiliation of Christian Toval-Ruiz changed from “Department of Microbiology and Parasitology, National Autonomous University of Nicaragua-León, León, Nicaragua” to “Universidad La Salle, León. Nicaragua.”
- Presence of other child using diapers was added to Table 3
- Figures S1 and S2 were included in the supplementary file, and the legends were added after reference.
- References were updated, and references 6-9, 25, 31, 33, and 34 were included.
- State: “The funders had no role in study design, data collection and analysis, decision to publish, or preparation of the manuscript” was added in the financial support section according to the journal's policy
